# Nonequilibrium strongly hyperuniform fluids of circle active particles with large local density fluctuations

**DOI:** 10.1126/sciadv.aau7423

**Published:** 2019-01-25

**Authors:** Qun-Li Lei, Massimo Pica Ciamarra, Ran Ni

**Affiliations:** 1 School of Chemical and Biomedical Engineering, Nanyang Technological University, 62 Nanyang Drive, Singapore 637459, Singapore.; 2 Division of Physics and Applied Physics, School of Physical and Mathematical Sciences, Nanyang Technological University, 21 Nanyang Link, Singapore 637371, Singapore.; 3 CNR-SPIN, Dipartimento di Scienze Fisiche, Università di Napoli Federico II, I-80126 Napoli, Italy.

## Abstract

Disordered hyperuniform structures are an exotic state of matter having vanishing long-wavelength density fluctuations similar to perfect crystals but without long-range order. Although its importance in materials science has been brought to the fore in past decades, the rational design of experimentally realizable disordered strongly hyperuniform microstructures remains challenging. Here we find a new type of nonequilibrium fluid with strong hyperuniformity in two-dimensional systems of chiral active particles, where particles perform independent circular motions of the radius *R* with the same handedness. This new hyperuniform fluid features a special length scale, i.e., the diameter of the circular trajectory of particles, below which large density fluctuations are observed. By developing a dynamic mean-field theory, we show that the large local density fluctuations can be explained as a motility-induced microphase separation, while the Fickian diffusion at large length scales and local center-of-mass-conserved noises are responsible for the global hyperuniformity.

## Teaser

Dynamic hierarchical structures with strong hyperuniformity are found in chiral active matter systems.

## INTRODUCTION

Perfectly ordered structures, such as crystals or quasi-crystals at zero temperature, are usually associated with some discrete symmetries and exhibit long-range correlations, leading to the structure factor of the system *S*(*q* → 0) = 0 (*1*). Similarly, the local density variance in these structures 〈δρ^2^〉 scales with the window size of observation *L* as 〈δρ^2^〉 ~ *L*^−λ^ with λ = *d* + 1, where *d* is the dimensionality of the system. In contrast, in normal disordered structures, e.g., conventional gases, liquids and amorphous solids, and even thermalized crytals, the long-wavelength density fluctuation makes *S*(*q* → 0) = const. > 0 and λ = *d*. Recently, the concept of hyperuniformity was introduced to study the state of matter (*2*). A structure is hyperuniform when it has vanishing long-wavelength density fluctuations, i.e., *S*(*q* → 0) ~ *q*^α^ → 0 with α > 0 and 〈δρ^2^〉 ~ *L*^−λ^ with *d* < λ ≤ *d* + 1 (*2*). It has been found in the past two decades that, besides the ordered hyperuniform structures, i.e., perfect crystals and quasi-crystals, a number of disordered structures are also hyperuniform, including the maximally random jammed packing (*3*), avian photoreceptor patterns (*4*), and some nonequilibrium systems (*5*–*10*).

Disordered hyperuniform structures have received an increasing amount of scientific attention, as some strongly hyperuniform disordered structures with λ = *d* + 1 exhibit similar properties as crystals with even better performance, e.g., large isotropic photonic bandgaps insensitive to defects (*11*) can be opened at low dielectric contrast (*12*). Although ideal hyperuniform structures similar to perfect crystals are unavoidably affected by thermal excitation, it still shows promise in designing robust disordered materials with novel functionalities (*1*, *13*–*16*). By far, various protocols were developed to design particle interactions to form disordered hyperuniform ground states in classical many-particle systems. However, the resulting interactions normally have delicate long-range or multibody terms (*1*), making their experimental realization highly challenging. An alternative approach is to use driven systems, e.g., self-organized colloidal suspensions under periodic shearing (*17*), to form nonequilibrium dynamic hyperuniform states, which can effectively avoid the dynamic trapping, and, in principle, have a higher resistance to thermal perturbations (*7*). Now, experimentalists only succeeded in generating weakly hyperuniform structures with λ ≃ *d* + 0.5 (*8*) using this method. Nevertheless, this is certainly a direction that deserves further investigation (*10*), as some self-driven systems, or active matter systems, have produced a number of unexpected emergent phenomena never found in corresponding equilibrium systems (*18*–*22*). However, in conventional active matter systems, i.e., active nematic systems and active Brownian particles systems, giant number fluctuations characterized by λ < *d* (*19*) and motility-induced phase separation (MIPS) with λ ≃ 0 (*20*–*23*) are usually observed. These large density fluctuations seemingly prohibit the formation of hyperuniform structures in active matter systems.

Recently, chiral active matter whose motion is chiral-symmetry broken, e.g., active particles/swimmers with circular motion in two-dimensional (2D) (*24*–*28*) or active spinner fluids (*29*), has attracted considerable attention. Both experiments and simulations have shown many interesting collective phenomena in these systems (*25*, *30*–*33*). In this work, using computer simulations combined with analytic theories, we study a 2D system of circle active particles, which perform independent circular motion with the same handedness and random circling phases. We show that in the limit of strong driving or zero thermal noise, with increasing density of particles or radius of circular motion *R*, the system undergoes an absorbing-active transition forming a nonequilibrium strongly hyperuniform fluid phase with density variance 〈δρ^2^〉 ~ *L*^−3^ (*L* → ∞) the same as in perfect crystals. Further increasing the density or *R* triggers the formation of dynamic clusters, which results in large local density fluctuations. These fluctuations are “confined” within the length scale of *R*, while the strong hyperuniformity persists at large length scales. This surprising coexistence of large local density fluctuations and the global hyperuniformity is explained by dynamic mean-field theories at different length scales.

## RESULTS

### Model

**Fig. 1:**
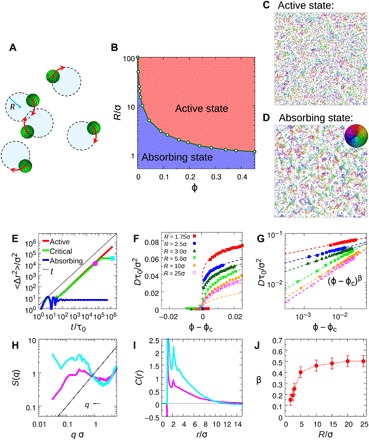
Absorbing-active transition. (**A**) Schematic of the 2D system of circle active particles. (**B**) Dynamic phase diagram in the representation of packing fraction ϕ and circle radius *R*. (**C** and **D**) Typical snapshots of the active and adsorbing states near the critical point with ϕ = 0.20 with *R* = 1.75σ, where the color indicates the self-propulsion orientation of each particle. These two states are marked as magenta and cyan solid symbols, respectively, in (E). (**E**) MSD as functions of time for system with *R* = 1.75σ started from random configurations. Red line, active state (ϕ = 0.22); blue line, absorbing state (ϕ = 0.01); green line, system near the critical point (ϕ = 0.20) in which the system ultimately falls into the absorbing state after a long simulation time. (**F** and **G**) Diffusion constant as functions of ϕ near the critical point ϕ_c_ for systems with different *R*. The dashed lines are the fitting of power law (ϕ − ϕ_c_)^β^. (**H** and **I**) The structure factor *S*(*q*) and the orientation correlation function *C*(*r*) of active (magenta) and absorbing (cyan) states as marked by solid symbols in (E). (**J**) The measured critical exponent β from (G) as a function of *R*. For all the calculations, *N* = 10,000 and *T*_R_ =0.

As illustrated in Fig. 1A, we consider a 2D suspension of *N* active colloidal particles with diameter σ. Each particle experiences an in-plane force *F*^p^ with random initial orientation and a constant torque Ω perpendicular to the plane, which drive the particles to perform circular motion with the same handiness (*30*, *31*). The dynamics of particle *i* at finite temperature *T* is governed by the over-damped Langevin equations (*26*, *30*)

$${\dot{r}}_{i}(t)=\gamma_{t}^{-1}[-\nabla_{i}U(t)+F^{p}e_{i}(t)]+\sqrt{2k_{B}T/\gamma_{t}}\xi_{i}^{t}(t)$$ (1)

$${\dot{e}}_{i}(t)=[\gamma_{r}^{-1}\Omega +\sqrt{2k_{B}T/\gamma_{r}}\xi_{i}^{r}(t)]\times e_{i}(t)$$ (2)

where **r**_*i*_ and **e**_*i*_ are the position of particle *i* and its self-propulsion orientation, respectively, and *k*_B_ is the Boltzmann constant. γ_t/r_ is the translational/rotational friction coefficient. For simplicity, we set γ_t_ = γ_r_/σ^2^. $\xi_{i}^{t}(t)$ and $\xi_{i}^{r}(t)$ represent Gaussian noises with zero mean and unit variance. We use Weeks-Chandler-Andersen (WCA) potential to mimic the excluded volume interaction between colloidal particles *U*(*t*) (see Methods). The packing fraction of the system is defined as ϕ = ρσ^2^π/4, where ρ is the particle density. The self-propulsion speed of particle is $v_{0}=\gamma_{t}^{-1}F^{p}$. The reduced noise strength in the system is defined as *T*_R_ = *k*_B_*T*/(*F*^p^σ) that measures the strength of thermal noise compared with the self-propulsion. In the zero noise limit, i.e., *T*_R_ = 0, isolated active particles perform circular motions with fixed radius *R* = *F*^p^σ^2^/Ω and period Γ = 2πγ_r_/Ω. This athermal noise-free situation is the major focus of this work, and the effect of thermal noise is discussed later.

### Absorbing-active transition

We first simulate systems with *N* = 10,000 and *T*_R_ = 0. Under low packing fraction ϕ and small *R* condition, we find that the system falls into an absorbing or arrested state, in which each particle performs an independent circular motion without collisions and the mean squared displacement (MSD) 〈Δ*r*^2^〉 of particles develops a plateau at long time (blue line in Fig. 1E). With increasing ϕ or *R*, the collisions between particles become more frequent, making the system unable to find a noninteracting state. Thus, the system remains at an active diffusive state with MSD 〈Δ*r*^2^〉 ~ 4*Dt* (*t* → ∞) (red line in Fig. 1E). Here, *D* is the long-time diffusion constant. The phase behaviors of the system are summarized in the phase diagram Fig. 1B. In the following, we focus on the absorbing-active transition close to the boundary of two phases.

In Fig. 1F, we plot *D* as a function of ϕ − ϕ_c_ for different *R*. Here, we obtain ϕ_c_ by fitting with the critical power law *D* ~ (ϕ − ϕ_c_)^β^, which determines the phase boundary in Fig. 1B. One can observe a sharp transition from the absorbing state [*D*(ϕ) = 0] to the active state [*D*(ϕ) > 0] when increasing ϕ at a small *R* = 1.75σ. The transition becomes smoother as *R* increases. In Fig. 1G, we show the log-log plot of *D* as a function of ϕ − ϕ_c_. The obtained slope β is given in Fig. 1J. We find that the critical exponent β is about 0.15 for *R* = 1.75σ, which is substantially smaller than the values in classical absorbing transitions, i.e., β = 0.58 for directed percolation and β = 0.64 for conserved directed percolation (Manna type) (*6*–*34*). Such a small critical exponent is independent of system size (see fig. S1). With increasing *R*, we find that β increases to around 0.5 for *R* > 10σ. A similar increase of the critical exponent with increasing interaction range (in our case, *R*) has been reported (*35*). In our system, ϕ_c_ would decrease to zero with increasing *R*. Hence, β at large *R* cannot be directly obtained in our system due to the divergence of simulation time needed at the dilute limit.

To understand the physics behind the absorbing-active transition at small *R*, we choose a packing fraction ϕ = 0.20 close to the critical point (ϕ_c_ = 0.195) for system with *R* = 1.75σ. The MSD for the system started from random configuration is shown by the green line in Fig. 1E, in which one can see that the system ultimately falls into the absorbing state after staying at the active state for a long time. In Fig. 1 (C and D), we show typical snapshots for the active state and absorbing state before and after the absorbing transition, as indicated by the magenta and cyan symbols in Fig. 1E. Movies for these two states can be found in movies S1 and S2. One can notice a marked structural difference between these two states. In the absorbing state, particles with similar orientation form finite synchronized clusters, while the active state is more homogeneous without noticeable spatial heterogeneity. This structural difference is also reflected in the structure factor *S*(*q*) and orientation correlation function *C*(*r*) = 〈∑_*i*≠*j*_ **e**_*i*_ ⋅ **e**_*j*_ δ(*r*_*ij*_ − *r*)〉/ρ*N*, as shown in Fig. 1 (H and I). Compared with the active state, *S*(*q*) for the absorbing state develops a pronounced peak at *q*σ ≃ 0.2, and the corresponding *C*(*r*) also shows a stronger orientation correlation. The synchronized clusters formation in our circle active particle system with isotropic circling-phase distribution shares a similar mechanism with the phase separation observed in an experimental bimodal phase-distributed system (see also fig. S4) (*26*). Both are a result of a crowding-induced attraction between particles with the same circling phase. We find that this distinct structural transformation during the absorbing transition is absent in the conventional absorbing transition (*5*, *6*, *17*, *34*), suggesting that the small critical exponent measured in our system has a structural origin. With increasing *R*, the structural difference between two phases becomes weaker (see fig. S2), which occurs simultaneously with the increase of β. Further studies combining with finite-size analysis are necessary for determining whether the absorbing-active transition at small *R* is first order.

### Hyperuniformity and large local density fluctuations

**Fig. 2:**
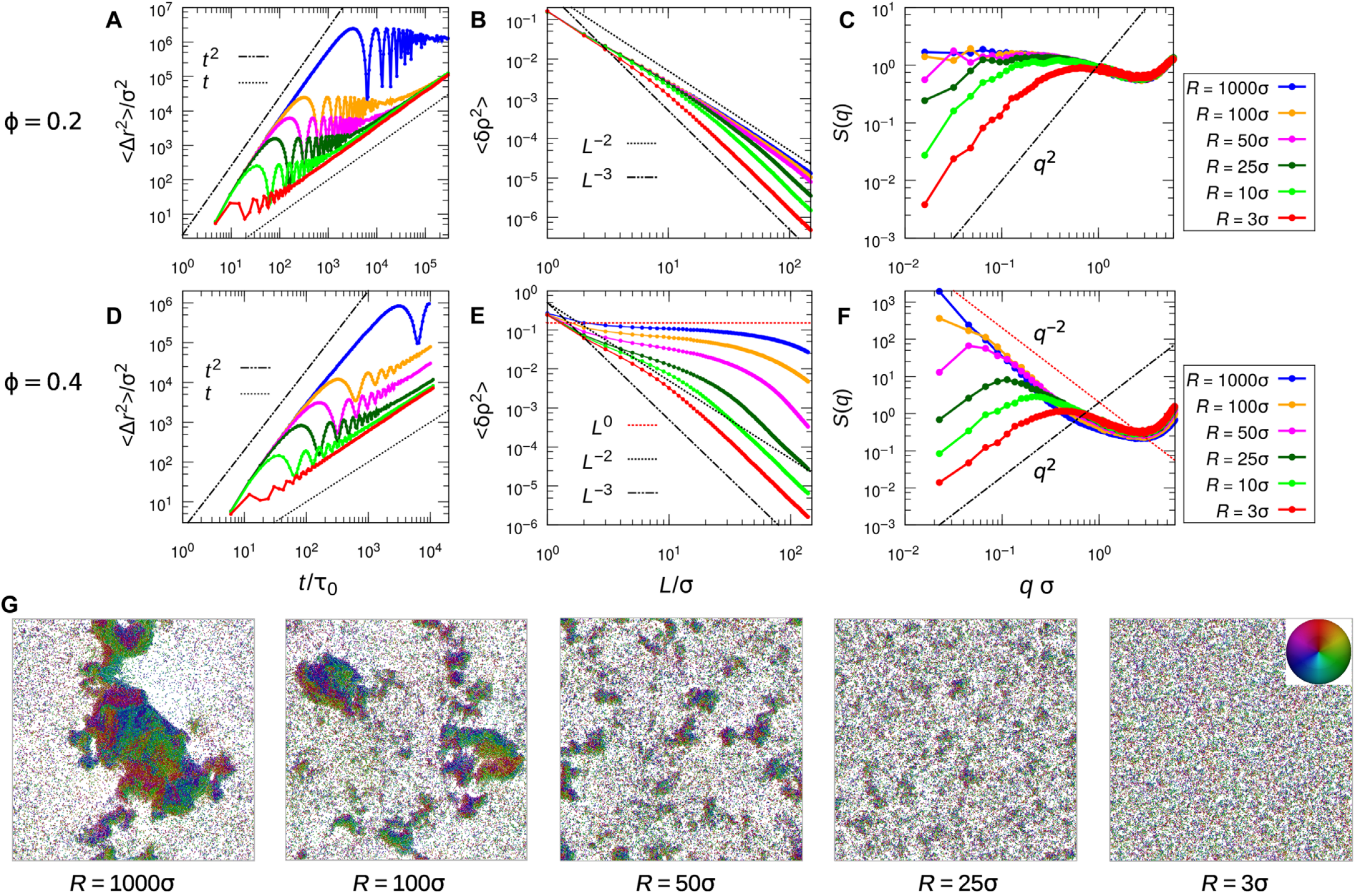
Dynamic hyperuniform state. (**A** and **D**) MSD as functions of *t* for various *R*. (**B** and **E**) Density variances 〈δρ^2^〉 as functions of window size *L* for various *R*. The *L*^−3^ asymptotic line indicates the hyperuniform scaling, which is the same as in perfect crystals. The *L*^−2^ scaling is for normal fluids, while *L*^0^ is for clustering- or phase separation–induced large density fluctuations. (**C** and **F**) Structure factor *S*(*q*) for various *R*. The *q*^2^ asymptotic line indicates the hyperuniform scaling, while the q^−2^ line represents clustering- or phase separation–induced large density fluctuations. (**G**) Typical snapshots for systems at ϕ = 0.4 with various *R*. For all the calculations, *N* = 40,000 and *T*_R_.

As shown in Fig. 1H, for the active state near the critical point in the system with *R* = 1.75σ, the structure factor of the system exhibits hyperuniform scaling *S*(*q*) ~ *q*(*q* → 0). In the random organization model aiming to mimic the colloidal suspension under periodic shearing, a similar hyperuniform scaling was observed near the critical point (*5*, *6*). However, for relative large *R*, the critical *q* scaling shifts to a faster *q*^2^ scaling (see fig. S2). To explore this further, we simulate a system of *N* = 40,000 circle active particles. In Fig. 2A, we first plot the MSD for active state systems with different *R* at ϕ = 0.2. We find that the diffusivity in the system rises slightly with increasing *R*. By further checking the scalings of the density variance 〈δρ^2^〉 and *S*(*q*) for different *R* (see Fig. 2, B and C), we observe a strong hyperuniformity in the systems with *R* ≤ 25σ, as indicated by the asymptotic behaviors: 〈δρ^2^〉 ~ *L*^−3^ (*L* → ∞) and *S*(*q*) ~ *q*^2^(*q* → 0). From 〈δρ^2^〉, one can identify an *R*-dependent length scale *L*_HU_, above which the system becomes hyperuniform, while below which the system behaves like normal fluids, i.e., 〈δρ^2^〉 ~ *L*^−2^ (Fig. 2B). In *S*(*q*), a similar threshold *q*_HU_ can be found, which features the end of the hyperuniform scaling. With increasing *R*, *L*_HU_ increases and *q*_HU_ decreases until *R* ≥ 50σ, where the hyperuniform scaling becomes less apparent due to the system finite-size effect as discussed below. This implies that *R* controls the length scales at which the system exhibits hyperuniformity.

Figure 2 (D to F) shows the result of an analogous investigation for higher-density systems with ϕ = 0.4. Compared with low-density systems, we observe pronounced enhancement of the diffusivity with increasing *R* (Fig. 2D). The high-density systems also show clear hyperuniform scaling at large length scales for *R* ≤ 50σ and the threshold *L*_HU_ (or *q*_HU_) increases (or decreases) with increasing *R* (Fig. 2, E and F). However, at ϕ = 0.4, we find 〈δρ^2^〉 ~ *L*^−λ^ with λ < 2 when *L* ≪ *L*_HU_ and λ decreases with larger *R*. This implies that at length scales *L* ≪ *L*_HU_, the system exhibits large density fluctuations, whose strength and length scale are both controlled by *R*. This large fluctuation can also be identified by the scaling *S*(*q*) ~ *q*^−2^ for *q* > *q*_HU_ as shown in Fig. 2F, which was reported as a signature of critical instability of active particle system undergoing MIPS (*20*). However, in most of our systems except *R* ≥ 100σ, *S*(*q*) ~ *q*^−2^ does not diverge at *q*_HU_ ≃ 0 as in MIPS (*20*) but stops at a finite *q*_HU_. This suggests that the large local density fluctuations observed in our system are due to clustering or microphase formation. The crossover of two different scalings, i.e., large density fluctuations and hyperuniformity, creates a peak in *S*(*q*) at *q*_HU_, whose height increases with larger *R*. Actually, for *R* ≥ 100σ, we speculate that in much larger systems, one can still observe the peak at finite *q*_HU_ for ϕ = 0.4 and the hyperuniform scaling for both ϕ = 0.2 and 0.4, as suggested by later theoretical analyses. In fig. S3, *S*(*q*) is shown for a larger system (*N* = 102,400) at ϕ = 0.4. The result agrees with our speculation. Typical snapshots of the system at ϕ = 0.4 with various *R* are shown in Fig. 2G (also in movies S3 to S7 and fig. S3), and one can see many finite-size clusters disappearing and reforming in the system. The average size of these dynamic clusters increases with increasing *R*, and at *R* = 1000σ, because of the finite-size effect, the clusters percolate the simulation box. These findings are intriguing, as hyperuniformity and large density fluctuations induced by dynamic cluster formation are two seemingly opposite phenomena, which coexist here in the same system at different length scales. In the following, we formulate dynamic mean-field theories to understand this new dynamic hyperuniform fluid with large local density fluctuations.

### Dynamic mean-field theory

Starting from the *N*-body Smoluchowski equation for active Brownian particles (*20*, *21*), one can prove (section S1) that in a homogeneous circle active particles system with vanishing orientation order parameter $Q=\langle ee^{T}-\frac{1}{2}1\rangle$, the time-dependent local density field ρ(**r**, *t*) and the local polarization field **p**(**r**, *t*) satisfy

$$\partial_{t}\rho =-\nabla \cdot [v_{e}(\rho )p-D_{e}\nabla \rho ]$$ (3)

$$\partial_{t}p=-\frac{1}{2}\nabla [v_{e}(\rho )\rho ]+D_{e}\nabla^{2}p+\Omega_{r}\times p$$ (4)

where *v*_e_(ρ) = *v*_0_ + ζρ is a density-dependent effective velocity of particles with a negative ζ reflecting the motility-induced “self-trapping” effect. *D*_e_ is the effective diffusion constant originated from the “evasive” motion of particles due to the collisions with neighboring particles (*21*). $\Omega_{r}=\gamma_{r}^{-1}\Omega$ is the reduced torque. The isotropic homogeneous state, i.e., $\left[\rho (r,t)=\bar{\rho },p(r,t)=0\right]$, is the solution to Eqs. 3 and 4. By making a weak perturbation around this state, i.e., $\left[\rho (r,t)=\bar{\rho }+\delta \rho (r,t),p(r,t)=\delta p(r,t)\right]$, we obtain two linearized equations in the Fourier space with the first-order approximation

$$(i\omega +D_{e}q^{2})\delta \rho_{q,\omega }=-iv_{e}q\cdot p_{q,\omega }$$ (5)

$$(i\omega +D_{e}q^{2})p_{q,\omega }=\Omega_{r}\times p_{q,\omega }-iwq\delta \rho_{q,\omega }$$ (6)

where [δρ_**q**,ω_, **p_q,ω_**] = ∫*d***r** ⋅ *e*^−*i***q**⋅**r**^∫*dt e*^−*i*ω*t*^[δρ, **p**], and $w=v_{0}/2+\zeta \bar{\rho }$ is the parameter indicating the strength of self-trapping effect. By solving Eqs. 5 and 6, one obtains the dispersion relationship, which includes a diffusive mode ω_0_ = *iD*_e_*q*^2^ and two nondiffusive modes

$$\omega_{1,2}=iD_{e}q^{2}\pm \sqrt{v_{e}wq^{2}+\Omega_{r}^{2}}$$ (7)

The growth rate of the mode is *κ* = *Re*(*i*ω), whose sign determines whether the perturbation δρ ~ *e*^*i*ω*t*+*i***q**⋅**r**^ grows or decays. One can prove that the mode 1 always decays, while the mode 2 may grow for *w* < 0 with

$$\kappa_{2}=\{\begin{array}{cc} -D_{e}q^{2} & q<\frac{v_{0}}{\sqrt{-v_{e}w}}R^{-1}\\ -D_{e}q^{2}+\sqrt{-v_{e}wq^{2}-\Omega_{r}^{2}} & q>\frac{v_{0}}{\sqrt{-v_{e}w}}R^{-1} \end{array}$$ (8)

**Fig. 3:**
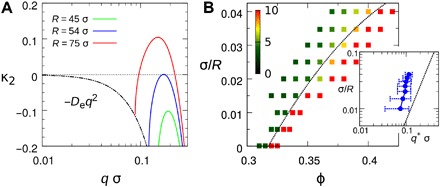
Dynamic microphase separation. (**A**) Growing rate κ_2_ as functions of *q* for systems at ϕ = 0.35 with various *R* obtained from the dynamic mean-field theory. (**B**) Measured heights of the first peak in *S*(*q*) for systems with different combinations of *R* and ϕ in computer simulations indicated by the color of the symbols. The dotted line is the fitting using Eq. 9 for the phase boundary. Inset shows the measured position of the first peak in *S*(*q*) in computer simulations (symbols) and the theoretical prediction (dotted line) based on the fitting in (B).

In Fig. 3A, we plot three typical situations of κ_2_ as functions of *q* at high density (*w* < 0) by varying *R* (or Ω_r_). One can see that for finite *R*, κ_2_ decreases from zero following the typical diffusive mode −*D*_e_*q*^2^ from *q* = 0 to $\frac{v_{0}}{\sqrt{-v_{e}w}}R^{-1}$, above which κ_2_ starts to increase and has the chance to be positive at finite *q* > 0. This implies that the homogeneous system becomes unstable as a result of the growing fluctuation of finite wavelength. In the mean-field picture, this typically indicates that the system undergoes a microphase separation. This scenario is different from the complete phase separation in which instability starts from the infinite wavelength at *q* = 0 (*20*, *21*).

The instability point of the system is defined as $\kappa_{2}^{\text{max}}(q*)=0$ for q* > 0, from which we can obtain the relationship between the critical packing fraction ϕ* and q* (see section S2)

$$(\frac{\phi *}{\phi_{c}}-1)(2-\frac{\phi *}{\phi_{c}})=\frac{{8D}_{e}}{v_{0}R}$$ (9)

$$q*=\sqrt{\frac{v_{0}}{{RD}_{e}}}$$ (10)

where ϕ_c_ ≃ 0.32 is the critical packing fraction for *R* = ∞ at which the simulation shows complete MIPS (q* = 0). This suggests that MIPS can be seen as a limit of the microphase separation in our system when *R* → ∞ at the mean-field level. For this special case, when ϕ approaches ϕ_c_, *S*(0) is supposed to jump from a finite value to ∞, which marks the “spinodal” of MIPS. For systems with finite *R*, the first peak of *S*(*q*), which is located around q*, is also expected to jump up to a higher value when the system crosses the “microphase separation point.” In Fig. 3B, we plot the measured heights of the first peak in *S*(*q*) for various combinations of *R* and ϕ in computer simulations. We find that when *R* > 50σ, the height of the first peak in *S*(*q*) jumps from below 5 to above 10 with increasing ϕ over ϕ*. The transition becomes smoother for systems of smaller *R*. Therefore, we choose *S*(*q*) = 7 as the threshold to fit the phase boundary using Eq. 9 (the dotted line in Fig. 3B), which leads to *D*_*e*_ ≃ 0.62*v*_0_*σ*. In the inset of Fig. 3B, we plot the location of q* measured in simulations and the prediction of Eq. 10 with the same *D*_e_. The simulation results agree almost quantitatively with the theoretical prediction. For small *R* cases (*R* < 50σ), there is a noticeable inconsistency, suggesting the possible breakdown of the mean-field theory. In these cases, the size of clusters in the system is comparable with the particle size. Therefore, microphase separation is no longer a proper concept to describe what happens in the system. Even for large *R* cases, which seem consistent with the mean-field microphase separation scenario, it still remains open whether there exist true sharp microphase separation points for finite *R* or if the sharpness of the transition diverges at *R* → ∞.

To summarize, this theoretical analysis rationalizes the instability in the circle active particle system as a result of motility-induced self-trapping effect (negative *w* in Eqs. 7 and 8). The existence of nonzero torque Ω_r_ restricts the growth of density fluctuations within a finite wavelength proportional to the circle radius *R*, which is the underlying reason for the dynamic clusters formation and the resulting large local density fluctuations observed in our simulations.

### Mechanism of global hyperuniformity

**Fig. 4:**
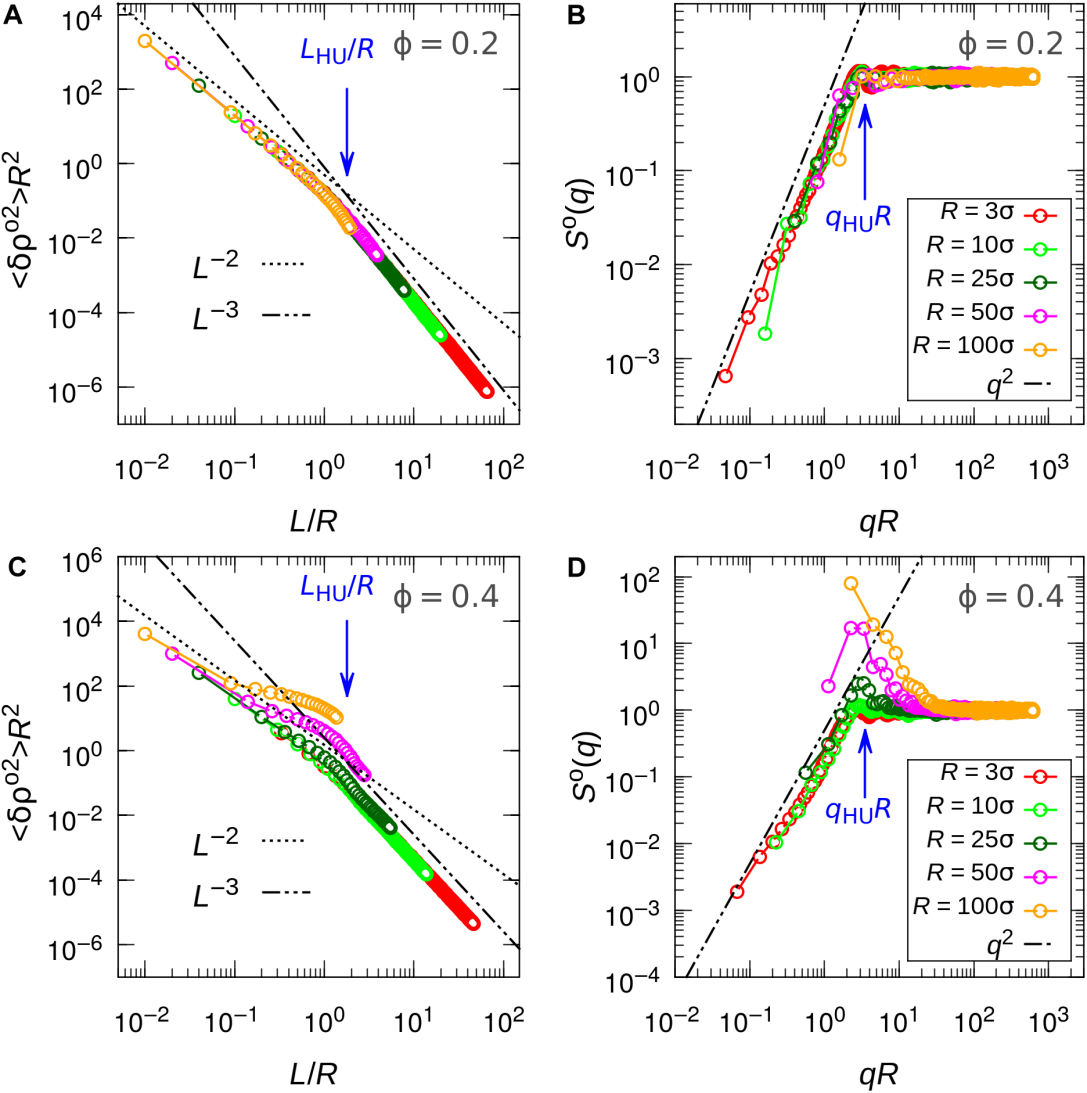
Global hyperuniformity. (**A** and **C**) Scaled density variance 〈δρ^o2^〉*R*^2^ as functions of *L*/*R* and (**B** and **D**) *S*^o^(*q*) as functions of *qR*, for various *R* at ϕ = 0.2 (A and B) and 0.4 (C and D), respectively.

To understand the hyperuniformity at large length scales, we focus on the spatial distribution of the instantaneous circling centers of active particles, which we treat as effective particles with radius *R*. For particle *i* at position **r**_*i*_, the circling center is at $r_{i}^{o}=r_{i}-\sigma^{2}(F_{i}^{p}\times \Omega )/{\left|\Omega \right|}^{2}$, which is a fixed position for an isolated circle active particle. The motion of these effective particles is due to collisions with other particles. This is similar to what happens in the random organization model (*5*, *6*, *10*), where only overlapped particles experience random kicks. As indicated in the previous theoretical analysis, the self-trapping and the growing density fluctuations are confined within the length scale of *R*. At larger length scales, fluctuations decay as the diffusive mode (Fig. 3A). These imply that the dynamic equation for the density field of these effective particles ρ^o^(**r**, *t*) at large length scales can be described by the Fick’s law of diffusion ∂_*t*_ρ = *D*∇^2^ρ in the *q* space

$$\partial_{t}\rho_{q}^{o}=-D_{e}^{o}q^{2}\rho_{q}^{o}+\xi_{q}(t)\;\text{for}\;(q\ll \frac{2\pi }{R})$$ (11)

with $\left[\rho_{q}^{o},\xi_{q}]=\int dr\cdot e^{-iq\cdot r}[\rho^{o},\xi \right]$. Here, $D_{e}^{o}$ is the diffusion coefficient of effective particles and ξ_**q**_(*t*) is an additional noise term. Since we neglect the thermal noise, ξ_**q**_(*t*) comes from the chaotic multiparticle interaction (*36*), which obeys the center of mass conservation (CMC). As proven in (*10*), the noise with additional CMC appears as a double-space derivative in the diffusion equation, i.e., $\xi (t)=\sqrt{\bar{\rho }}\nabla^{2}\eta (t)$, with 〈η(**r**, *t*)η(**r**′, *t*′)〉 = *A*^2^δ(**r** − **r**′)δ(*t* − *t*′) and *A* as the strength of the noise. This is different from the conventional single-space derivative noise that arises solely from the particle number conservation (*18*, *37*). Following (*10*), by adding such a double-space derivative noise term into Eq. 11 as the perturbation, one obtains the dynamic equation for the density fluctuation δρ^o^ in the Fourier space

$$i\omega \delta \rho_{q,\omega }^{o}=-D_{e}^{o}q^{2}\delta \rho_{q,\omega }^{o}-q^{2}\sqrt{\bar{\rho }}\eta_{q,\omega }\;\text{for}\;(q\ll \frac{2\pi }{R})$$ (12)

where η_**q**,ω_ is the Fourier transform of the noise η(**r**, *t*). From Eq. 12, we obtain the structure factor

$$\begin{array}{c} S^{o}(q)=\int_{-\infty }^{\infty }\frac{1}{2\pi \tau_{\text{max}}N}\langle \delta \rho_{q,\omega }^{o}\delta \rho_{q,\omega }^{o*}\rangle d\omega \\ =\frac{A^{2}}{{2D}_{e}}q^{2}\;\text{for}\;(q\ll \frac{2\pi }{R}) \end{array}$$ (13)

where τ_max_ is the maximum observation time (see section S3). Equation 13 shows the same hyperuniform exponent in *S*(*q*) as observed in the simulations and also shows the strongest hyperuniform scaling for density variance 〈δρ^o2^〉 ~ *L*^−3^ at the length scales larger than *R* (*2*). In Fig. 4A, we plot the scaled density variance 〈δρ^o2^〉*R*^2^ versus *L*/*R* for systems with various *R* at ϕ = 0.2, and one can see all points collapse into a single curve. The curve consists of two distinct scalings with a strongly hyperuniform scaling *L*^−3^ at *L* > *L*_HU_ ≃ 2*R* and a normal fluid-like scaling *L*^−2^ at *L* < *L*_HU_. Similar collapse is shown in Fig. 4B for *S*^o^(*q*) versus *qR*, where the hyperuniform scaling *S*^o^(*q*) ~ *q*^2^ stops at *q*_HU_ ≃ 2π/*L*_HU_. For high-density systems, i.e., ϕ = 0.4, we obtain the same crossover length scale *L*_HU_ ≃ 2*R* as shown in Fig. 4 (C and D). In this case, the density variance and *S*^o^(*q*) for different *R* do not collapse into a single curve because of cluster formation–induced large density fluctuations at *L* ≲ *L*_HU_. After obtaining the hyperuniform scaling for these effective particles, one can prove the existence of the same hyperuniform scaling at similar length scales for the circle active particles (*38*).

From these analyses, one can see that, in systems of circle active particles, there are two ingredients for the global hyperuniformity: (i) The circular trajectories of active particles localize their active motions, creating the Fickian diffusion condition for the active particles at large length scales; and (ii) the local density fluctuations originate from the chaotic multiparticle interactions, which obey the CMC. These arguments are based on (*10*), which produces the same hyperuniform scaling in *S*(*q*) using the random-organization model with CMC. Although the two systems share the similar mechanism of hyperuniformity at large length scales, there are also marked differences between them. First, the driving force in our system is persistent, and the dynamics of the active particles is overdamped and deterministic. Second, our system has a characteristic length *R*, which separates two completely different fluctuations. These cannot be described by the model in (*10*).

We notice that the two ingredients for the dynamic hyperuniformity above seem to be satisfied in a recent experimental system, in which spherical Janus colloids with additional soft repulsion perform almost perfect circling motion with the same chirality but two different phases, driven by external fields (*26*). The thermal noise was believed to be negligible in this system, and the interactions between these nearly athermal active particles produce an effective temperature, which controls both kinetic and phase behavior of the system (*26*). To test whether the hyperuniformity can exist in this circle active particle system with the bimodal circling-phase distribution, we simulate a low density system (ϕ = 0.2) with two different circle radii *R* = 1σ and 3σ using the model (see Methods), which produced almost the same experimental result in (*26*). Our result is shown in fig. S4, from which we find the bimodal-distributed system falls into a phase-separated absorbing state for *R* = 1σ but stays in an active mixing (lane) state for *R* = 3σ, consistent with the previous finding (*26*). In the active mixing state, we observe the same hyperuniform scaling *S*(*q* → 0) ~ *q*^2^. This result demonstrates the robustness of the hyperuniformity mechanism unveiled by this general model and suggests a high possibility of realization in experiments. Moreover, it also suggests that other active systems satisfying the two ingredients, such as active spinner systems (*29*) or size/interaction oscillating particle systems (*39*), may be used to produce the same hyperuniformity as well.

### Effect of thermal noise

**Fig. 5:**
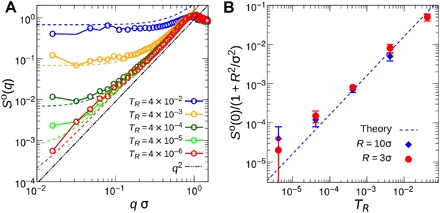
Effect of thermal noises on hyperuniformity. (**A**) Structure factor *S*^o^(*q*) under different reduced noise strength *T*_R_ for ϕ = 0.2 and *R* = 3σ. Open symbols show the simulation data, while the dashed lines are the theoretical predictions of Eq. 16. (**B**) Normalized *S*^o^(0) as functions of *T*_R_ from theoretical prediction (dashed line) and the fitting of simulation results (solid symbols) for systems with ϕ = 0.2. For all the calculations, *n* = 40,000.

According to the definition, a perfectly hyperuniform state requires *S*(0) to exactly equal zero. However, in real experimental systems, thermal noise is unavoidable. On the basis of the fluctuation-compressibility relationship

$$S(0)=\kappa_{T}\rho k_{B}T$$ (14)

any thermal equilibrated systems with the positive isothermal compressibility κ_T_ at finite temperatures cannot be strictly hyperuniform due to thermal excitation (*13*). In crystals, thermal excitation appears as phonon modes, which cause background scattering or thermal diffuse scattering, whose effect can be measured by the Debye-Waller factor (*13*, *40*). Nevertheless, the wide application of crystal materials indicates that thermalization only weakens but does not destroy most physical properties of the ground-state crystal. Similarly, near-hyperuniformity (*1*, *13*, *15*) in disordered structures may also be enough to achieve some desired functions, e.g., isotropic photonic/electronic bandgaps (*14*, *15*). In Fig. 5, we analyze the influence of thermal excitation on the ground-state hyperuniform system with ϕ = 0.2 and *R* = 3σ. We find that with a gradual increase of the reduced noise strength *T*_R_ from zero, *S*(*q* → 0) begins to saturate at some nonzero value, which increases along with *T*_R_ (Fig. 5A). In section S4, we introduce the thermal noise **f** = [*f*_*x*_, *f*_*y*_] in Eq. 11 as

$$\xi (t)=\sqrt{\bar{\rho }}\nabla \cdot [\nabla \eta (t)+f(t)]$$ (15)

where 〈*f*_*i*_(**r**, *t*)*f*_*j*_(**r**′, *t*′)〉 = 2*D*_therm_δ_*ij*_δ(**r** − **r**′)δ(*t* − *t*′) with $D_{\text{therm}}=k_{B}T\gamma_{t}^{-1}(1+R^{2}/\sigma^{2})$ the self-diffusion constant of effective particles due to the thermal Brownian motion (*25*). Here, we assume that the thermal noise is a first-order weak perturbation on the chaotic noise η(*t*), which leads to the decoupling of these two noise sources: 〈*f*_*i*_(**r**, *t*)η(**r**′, *t*′)〉 = 0. Then, we can estimate *S*^o^(*q*) as a function of the reduced noise strength *T*_R_ for low-density systems as

$$S^{o}(q)=\frac{v_{0}\sigma }{D_{e}}(1+\frac{R^{2}}{\sigma^{2}})T_{R}+\frac{A^{2}}{{2D}_{e}}q^{2}\;\text{for}\;(q<q_{\text{HU}})$$ (16)

In Fig. 5A, we plot the theoretical prediction of Eq. 16 as dashed lines for different *T*_R_ values at *R* = 3σ and ϕ = 0.2, by assuming them all across the same *S*^o^(*q*_HU_) point. In Fig. 5B, we compare the *S*^o^(0) from the theoretical prediction with the *S*^o^(0) obtained from the fitting of simulation results (see Methods) for systems with *R* = 3σ and 10σ at ϕ = 0.2. One can find quantitative agreements between simulation and theoretical predictions in Fig. 5, which suggest that the susceptibility of hyperuniformity to the noise in our nonequilibrium system is similar to that in thermalized crystals, i.e., Eq. 14. However, we also emphasize the difference: In our nonequilibrium system, the saturated value of *S*^o^(0) is determined by the driving force as well. Therefore, in experiments, it is possible to observe a large range of hyperuniform scaling in *S*(*q*) or density variance as long as the driving force is much larger than the thermal noise, i.e., *T*_R_ ≪ 1.

## DISCUSSION

In conclusion, by combining computer simulations with theoretical analyses, we investigate the dynamic phase behaviors in 2D systems of circle active particles. In the zero-noise limit, we find that with increasing the density of system or the radius of circular motion *R*, the system undergoes a transition from an absorbing state to an active fluid state, which is accompanied by a structural transformation for small *R*. In the active fluid state, we find a characteristic length scale *L*_HU_. For *L* ≫ *L*_HU_, the system exhibits strong hyperuniformity with the density variance scaling the same as in perfect crystals, while for *L* ≲ *L*_HU_, we observe normal random fluctuations at low density and large density fluctuations (cluster formation) at high density. To understand the mechanism of the phase behaviors of the system, we develop a dynamic mean-field theory. Linear stability analysis suggests that at the mean-field level, the large local density fluctuations at relative large *R* are a result of motility-induced microphase separation, which is confined within the length scale of *R*. For the global hyperuniformity, we attribute it to the interplay between the Fickian diffusion of active particles at large length scales and local particle collisions that conserve the center of mass (*10*). Our work demonstrates that two extreme fluctuations, i.e., large density fluctuations and hyperuniformity, can coexist in the same dynamic system at different length scales. We emphasize that this stable hierarchical hyperuniform fluid is conceptually different from the disordered hyperuniform solid or critical hyperuniform state. From a practical point of view, our results suggest that even for exotic disordered hyperuniform structures, there is still plenty of room at the “bottom,” i.e., one may construct arbitrary local complex structures (ordered or disordered) with extra functionalities without harming the global hyperuniformity. This provides large freedom in designing hierarchical disordered hyperuniform materials with unconventional properties.

## METHODS

In our simulations, we used a square simulation box with periodic boundary conditions in all directions, starting from initial configurations with random particle positions and orientations to make sure the initial structure factor *S*(*q*) ~ 1. The time unit was chosen to be the time that a particle moves a distance of σ in the dilute limit, i.e., τ_0_ = σ/*v*_0_. To mimic the excluded volume interaction between colloidal particles *i* and *j*, we used the WCA potential

$$U(r_{ij})=\{\begin{array}{cc} 4\varepsilon [{\left(\frac{\sigma }{r_{ij}}\right)}^{12}-{\left(\frac{\sigma }{r_{ij}}\right)}^{6}+\frac{1}{4}] & \left(r_{ij}<2^{1/6}\sigma \right)\\ 0 & \left(r_{ij}>2^{1/6}\sigma \right) \end{array}$$ (17)

where *r*_*ij*_ is the center to center distance between particles *i* and *j*, with σ as the diameter of particles. We chose ε = *F*^p^σ/24, which gives the typical contact distance *r*_c_ = σ between particles based on force balance $F^{p}={\frac{\partial U(r_{ij})}{{\partial r}_{ij}}|}_{r_{ij}=r_{c}}$. For systems at *T*_R_ = 0, to exclude the noise generated by discrete dynamic integrations, we used perfect convex polygons to approximate the closed circle trajectories of active particles. This is realized by finely tuning the integration step, which is around 10^−3^τ_0_.

For systems with bimodal distributed circling-phase, following (*26*), we added an additional soft repulsion *U*_s_ = *A* (*r*/σ)^−4^ with cutoff distance *r*_sc_ = 5σ and *A* = 7.5ε to model the isotropic dipole interaction between active particles. The self-propulsion force for this system was reset to *F*^p^ = 2^7/6^*A*/σ to make the dipolar interaction balance the driving force at *r*_c_ = 2^1/6^σ (*26*).

The density variance 〈δρ^2^〉 of the system was calculated using a spherical window whose diameter is smaller than the half of simulation box to avoid the finite-size effect. Under the periodic boundary condition, the structure factor *S*(*q*) was calculated for some discrete **q** vectors, i.e., $\left[q_{x},q_{y}]=\frac{2\pi }{L_{0}}[i,j](i,j=1,2,3\ldots \right)$, with *L*_0_ as the size of cubic simulation box. The simulation time for the equilibrating and sampling processes depends on the system size and density. For hyperuniform system, the criterion for the system to reach equilibrium is whether the hyperuniform scaling of *S*(*q*) has fully extended to the smallest *q*_min_ = 2π/*L*_0_ without further change. The fitting function used in Fig. 5B is Eq. 16, with an adjustable *T*_R_.

## Supplementary Material

http://advances.sciencemag.org/cgi/content/full/5/1/eaau7423/DC1

## SUPPLEMENTARY MATERIALS

Supplementary material for this article is available at http://advances.sciencemag.org/cgi/content/full/5/1/eaau7423/DC1 Section S1. Derivation of the dynamic mean-field theory for 2D system of circle active particles. Section S2. Linear stability analysis. Section S3. Calculation of *S*^o^(*q*) for system with CMC. Section S4. Effect of thermal noise on *S*^o^(*q*). Fig. S1. Finite-size effect on the long-time diffusion coefficient *D* as a function of packing fraction ϕ for systems with *R* = 1.75σ. Fig. S2. Structural comparison of active and absorbing state at *R* = 10σ near the critical point ϕ_c_ = 0.0194. Fig. S3. Structure factor *S*(*q*) for large systems with different *R* at φ = 0.4 and *T*_R_ = 0. Fig. S4. Hyperunifomity in an experimentally realizable system (*26*) with bimodal circling-phase distribution. Movie S1. Active state in Fig. 1C. Movie S2. Absorbing state in Fig. 1D. Movie S3. Active state with *R* = 1000σ in Fig. 2G. Movie S4. Active state with *R* = 100σ in Fig. 2G. Movie S5. Active state with *R* = 50σ in Fig. 2G. Movie S6. Active state with *R* = 25σ in Fig. 2G. Movie S7. Active state with *R* = 23σ in Fig. 2G.
