# Supplementary material for: Nonequilibrium strongly hyperuniform fluids of circle active particles with large local density fluctuations
Source: Sci Adv. 2019 Jan 25;5(1):eaau7423. doi: 10.1126/sciadv.aau7423 (PMC6357732; doi:10.1126/sciadv.aau7423)
Supplement: http://advances.sciencemag.org/cgi/content/full/5/1/eaau7423/DC1 [file supp_5_1_eaau7423__index.html]

Science Advances | Science Advances

## Supplementary Materials

**The PDF file includes:**

- Section S1. Derivation of the dynamic mean-field theory for 2D system of circle active particles.
- Section S2. Linear stability analysis.
- Section S3. Calculation of *S*o(*q) for system with CMC.*
- *Section S4. Effect of thermal noise on *S*o(*q*).*
- *Fig. S1. Finite-size effect on the long-time diffusion coefficient *D* as a function of packing fraction ϕ for systems with *R* = 1.75σ.*
- *Fig. S2. Structural comparison of active and absorbing state at *R* = 10σ near the critical point ϕc = 0.0194.*
- *Fig. S3. Structure factor *S*(*q*) for large systems with different *R* at φ = 0.4 and *T*R = 0.*
- *Fig. S4. Hyperunifomity in an experimentally realizable system (*26*) with bimodal circling-phase distribution.*

*Download PDF*

***Other Supplementary Material for this manuscript includes the following:***

- *Movie S1 (.mp4 format). Active state in Fig. 1C.*
- *Movie S2 (.mp4 format). Absorbing state in Fig. 1D.*
- *Movie S3 (.mp4 format). Active state with *R* = 1000σ in Fig. 2G.*
- *Movie S4 (.mp4 format). Active state with *R* = 100σ in Fig. 2G.*
- *Movie S5 (.mp4 format). Active state with *R* = 50σ in Fig. 2G.*
- *Movie S6 (.mp4 format). Active state with *R* = 25σ in Fig. 2G.*
- *Movie S7 (.mp4 format). Active state with *R* = 23σ in Fig. 2G.*

***Files in this Data Supplement:***

- *Adobe PDF - aau7423\_SM.pdf*
