# Supplementary material for: Nonequilibrium strongly hyperuniform fluids of circle active particles with large local density fluctuations
Source: Sci Adv. 2019 Jan 25;5(1):eaau7423. doi: 10.1126/sciadv.aau7423 (PMC6357732; doi:10.1126/sciadv.aau7423)
Supplement: http://advances.sciencemag.org/cgi/content/full/5/1/eaau7423/DC1 [file aau7423_SM.pdf]

Science Advances
AAAS

advances.sciencemag.org/cgi/content/full/5/1/eaau7423/DC1

Supplementary Materials for

Nonequilibrium strongly hyperuniform fluids of circle active particles with large local density fluctuations

Qun-Li Lei, Massimo Pica Ciamarra*, Ran Ni*

*Corresponding author. Email: massimo@ntu.edu.sg (M.P.C.); r.ni@ntu.edu.sg (R.N.)

Published 25 January 2019, *Sci. Adv.* **5**, eaau7423 (2019)
DOI: 10.1126/sciadv.aau7423

The PDF file includes:

Section S1. Derivation of the dynamic mean-field theory for 2D system of circle active particles
Section S2. Linear stability analysis
Section S3. Calculation of $S^{o}(q)$ for system with CMC
Section S4. Effect of thermal noise on $S^{o}(q)$
Fig. S1. Finite-size effect on the long-time diffusion coefficient D as a function of packing fraction ϕ for systems with $R = 1.75\sigma$.
Fig. S2. Structural comparison of active and absorbing state at $R = 10\sigma$ near the critical point $\phi_c = 0.0194$.
Fig. S3. Structure factor $S(q)$ for large systems with different R at $\phi = 0.4$ and $T_R = 0$.
Fig. S4. Hyperunifomity in an experimentally realizable system (*26*) with bimodal circling-phase distribution.

Other Supplementary Material for this manuscript includes the following:

(available at advances.sciencemag.org/cgi/content/full/5/1/eaau7423/DC1)

Movie S1 (.mp4 format). Active state in Fig. 1C.
Movie S2 (.mp4 format). Absorbing state in Fig. 1D.
Movie S3 (.mp4 format). Active state with $R = 1000\sigma$ in Fig. 2G.
Movie S4 (.mp4 format). Active state with $R = 100\sigma$ in Fig. 2G.
Movie S5 (.mp4 format). Active state with $R = 50\sigma$ in Fig. 2G.
Movie S6 (.mp4 format). Active state with $R = 25\sigma$ in Fig. 2G.
Movie S7 (.mp4 format). Active state with $R = 3\sigma$ in Fig. 2G.

Section S1. Derivation of the dynamic mean-field theory for 2D system of circle active particles

The theory starts with the joint probability $\psi_N(\{\mathbf{r}_i,\theta_i\},t)$ for N particles' position $\{\mathbf{r}_i\}$ and self-propulsion orientation $\{\theta_i\}$ with $i = 1,2,3\cdots N$ being the particle index. ψ_N satisfies the Smoluchowski equation (*20*, *21*)

$$\partial_t\psi_N = \sum_{i=1}^{N}\left\{\nabla_i\cdot[\gamma_t^{-1}\nabla_i U_N(t) - v_0\mathbf{e}_i + D_t\nabla_i] - \Omega_r\frac{\partial}{\partial\theta_i} + D_r\frac{\partial^2}{\partial^2\theta_i}\right\}\psi_N \tag{S1}$$

where U_N is the potential of the system; $\mathbf{e}_i = [\cos\theta_i, \sin\theta_i]$ is the direction of propulsion for particle i, with v_0 the propulsion speed; $\Omega_r = |\mathbf{\Omega}|/\gamma_r = 2\pi/\Gamma$ is the reduced torque. $D_{t,r} = k_BT/\gamma_{t,r}$ is the translational/rotational diffusion constant. Generally, one can prove that the reduced one-particle density $\psi_1(\mathbf{r}_1,\theta_1,t) = N\int d\mathbf{r}_2\cdots d\mathbf{r}_N\int d\theta_2\cdots d\theta_N\psi_N$ satisfies

$$\partial_t\psi_1 = \nabla_1\cdot[\gamma_t^{-1}\mathbf{F}_1 - v_0\mathbf{e}_1 + D_t\nabla_1]\psi_1 - \Omega_r\frac{\partial\psi_1}{\partial\theta_1} + D_r\frac{\partial^2\psi_1}{\partial^2\theta_1} \tag{S2}$$

where $\mathbf{F}_1$ is the force on a single active particle. With a mean-field approximation (*21*), $\mathbf{F}_1$ can be written as

$$\mathbf{F}_1\psi_1 = F_f\mathbf{e}_1\psi_1 + \gamma_t D_e\nabla_1\psi_1 \tag{S3}$$

where $F_f = \zeta\gamma_t\rho$ can be viewed as a density-dependent friction force as a result of the block from homogenously distributed neighboring particles. This force acts in the opposite direction of the self-propulsion forces, as reflected by the negative parameter ζ. The second term in Eq. (S3) describes the imbalanced force exerted on the active particle by its neighbors, which is a function of the density gradient. This force leads to the 'evasive' motion of the active particles and produces an effective diffusion constant D_e (*21*). Formally, Eq. (S3) can also be regarded as the expansion of $\mathbf{F}_1$ to the second order. Omitting the subscript in $\nabla_1, \psi_1, \theta_1, \mathbf{e}_1$ one can simplify Eq. (S3) into

$$\partial_t\psi = -\nabla\cdot[v_e(\rho)\mathbf{e}\psi - D_e'\nabla\psi] - \Omega_r\frac{\partial\psi}{\partial\theta} + D_r\frac{\partial^2\psi}{\partial^2\theta} \tag{S4}$$

with a density-dependent propulsion velocity $v_e(\rho) = v_0 + \zeta\rho$, which reflects the motility-induced "self-trapping" effect. The new effective diffusion constant becomes $D_e' = D_e + D_t$. From Eq. (S4), one can derive the dynamic equations for the local density $\rho(\mathbf{r},t) = \int d\theta\psi(\mathbf{r},\theta,t)$ and the local polarization field $\mathbf{p}(\mathbf{r},t) = \int d\theta\mathbf{e}\psi(\mathbf{r},\theta,t)$ by assuming a vanishing local orientational order parameter $\mathbf{Q} =< \mathbf{e}\mathbf{e}^T - \frac{1}{2}\mathbf{1} >$ (*20*, *21*) in the homogeneous state

$$\begin{aligned}\partial_t\rho(\mathbf{r},t) &= \int d\theta\,\partial_t\psi(\mathbf{r},t)\\ &= \int d\theta\left\{-\nabla\cdot[v_e(\rho)\mathbf{e}\psi - D_e'\nabla\psi] - \Omega_r\frac{\partial\psi}{\partial\theta} + D_r\frac{\partial^2\psi}{\partial^2\theta}\right\}\\ &= -\nabla\cdot\left[v_e(\rho)\int d\theta\mathbf{e}\psi - D_e'\nabla\int d\theta\psi\right] - \int\left\{\Omega_r\frac{\partial\psi}{\partial\theta} + D_r\frac{\partial^2\psi}{\partial^2\theta}\right\}d\theta\\ &= -\nabla\cdot[v_e(\rho)\mathbf{p} - D_e'\nabla\rho]\end{aligned} \tag{S5}$$

$$\begin{aligned}\partial_t\mathbf{p}(\mathbf{r},t) &= \int d\theta\mathbf{e}\,\partial_t\psi(\mathbf{r},\theta,t)\\ &= \int d\theta\mathbf{e}\left\{-\nabla\cdot[v_e(\rho)\mathbf{e}\psi - D_e'\nabla\psi_1] - \Omega_r\frac{\partial\psi}{\partial\theta} + D_r\frac{\partial^2\psi}{\partial^2\theta}\right\}\\ &= -\int d\theta\mathbf{e}\nabla\cdot\mathbf{e}\,v_e(\rho)\psi + D_e'\nabla^2\int d\theta\mathbf{e}\psi - \Omega_r\int d\theta\frac{\partial\psi}{\partial\theta}\mathbf{e} + D_r\int d\theta\frac{\partial^2\psi}{\partial^2\theta}\mathbf{e}\end{aligned}$$

$$= -\int d\theta \nabla[\mathbf{e}\mathbf{e}^T v_e(\rho)\psi] + D_e'\nabla^2\mathbf{p} - \Omega_r \int d\theta \left[\frac{\partial}{\partial\theta}\mathbf{e}\psi - \psi\frac{\partial \mathbf{e}}{\partial\theta}\right] + D_r \int d\theta \left[\frac{\partial}{\partial\theta}\left(\mathbf{e}\frac{\partial\psi}{\partial\theta}\right) - \frac{\partial}{\partial\theta}\left(\psi\frac{\partial \mathbf{e}}{\partial\theta}\right) + \psi\frac{\partial^2\mathbf{e}}{\partial^2\theta}\right]$$

$$= -\nabla\left[v_e(\rho)\int d\theta\left(\mathbf{e}\mathbf{e}^T - \frac{1}{2}\mathbf{1}\right)\psi\right] - \frac{1}{2}\nabla\left[v_e(\rho)\int d\theta\psi\right] + D_e'\nabla^2\mathbf{p} + \int d\theta \boldsymbol{\Omega}_r \times \mathbf{e}\psi - D_r\int d\theta \mathbf{e}\psi$$

$$= -\frac{1}{2}\nabla[v_e(\rho)\rho] + D_e'\nabla^2\mathbf{p} + \boldsymbol{\Omega}_r \times \mathbf{p} - D_r\mathbf{p} \quad \text{(S6)}$$

In the strong propulsion or zero thermal noise limit, i.e., $D_e \gg D_t$ and $|\boldsymbol{\Omega}_r| \gg D_r$, Eq. (S5, S6) can be simplified to

$$\partial_t\rho = -\nabla\cdot[v_e(\rho)\mathbf{p} - D_e\nabla\rho], \quad \text{(S7)}$$

$$\partial_t\mathbf{p} = -\frac{1}{2}\nabla[v_e(\rho)\rho] + D_e\nabla^2\mathbf{p} + \boldsymbol{\Omega}_r \times \mathbf{p} \quad \text{(S8)}$$

Section S2. Linear stability analysis

The homogeneous state $[\rho(\mathbf{r},t) = \overline{\rho}, \mathbf{p}(\mathbf{r},t) = 0]$ is a solution to Eq. (S7, S8). By taking a weak perturbation round this state, i.e., $[\rho(\mathbf{r},t) = \overline{\rho} + \delta\rho(\mathbf{r},t), \mathbf{p}(\mathbf{r},t) = \delta\mathbf{p}(\mathbf{r},t)]$, we obtain a set of linearized equations in the Fourier space with the first-order approximation

$$[i\omega + D_e q^2]\delta\rho_{\mathbf{q},\omega} = -iv_e\left(q_x \mathrm{p}^x_{\mathbf{q},\omega} + q_y \mathrm{p}^y_{\mathbf{q},\omega}\right) \quad \text{(S9)}$$
$$[i\omega + D_e q^2]\mathrm{p}^x_{\mathbf{q},\omega} = -\Omega_r \mathrm{p}^y_{\mathbf{q},\omega} - i\mathrm{w}q_x\delta\rho_{\mathbf{q},\omega} \quad \text{(S10)}$$
$$[i\omega + D_e q^2]\mathrm{p}^y_{\mathbf{q},\omega} = \Omega_r \mathrm{p}^x_{\mathbf{q},\omega} - i\mathrm{w}q_y\delta\rho_{\mathbf{q},\omega} \quad \text{(S11)}$$

where $[\delta\rho_{\mathrm{q},\omega}, \mathbf{p}_{\mathrm{q},\omega}] = \int d\mathbf{r}e^{-i\mathbf{q}\cdot\mathbf{r}}\int dte^{-i\omega t}[\delta\rho, \mathbf{p}]$ and $\mathrm{w} = \frac{1}{2}\left(v_e(\overline{\rho}) + \overline{\rho}\frac{\partial v_e}{\partial\rho}\Big|_{\overline{\rho}}\right) = v_0/2 + \zeta\rho$ is the parameter indicating the strength of self-trapping effect. By solving Eq. (S9-S11), one obtains the dispersion relationship of the system

$$(i\omega + D_e q^2)[(i\omega + D_e q^2)^2 + v_e\mathrm{w}q^2 + \Omega_r^2] = 0 \quad \text{(S12)}$$

Except for the diffusive mode $\omega_0 = iD_e q^2$, Eq. (S12) suggests two other non-diffusive modes the specific q

$$\omega_1 = iD_e q^2 + \sqrt{v_e\mathrm{w}q^2 + \Omega_r^2} \quad \text{(S13)}$$

$$\omega_2 = iD_e q^2 - \sqrt{v_e\mathrm{w}q^2 + \Omega_r^2} \quad \text{(S14)}$$

The growth rate of the mode is $\kappa = \mathrm{Re}(i\omega)$, whose sign determines whether the perturbation $\delta\rho \sim e^{i\omega t + i\mathbf{q}\cdot\mathbf{r}}$ grows or decays. Since w can change sign, κ is a conditional function. Nevertheless, one can prove that mode 1 always decays, while mode 2 decays for w > 0, but may grow for w < 0

$$\kappa_2 \sim \begin{cases} -D_e q^2 & q < \dfrac{v_0}{\sqrt{-v_e\mathrm{w}}}R^{-1} \\ -D_e q^2 + \sqrt{-v_e\mathrm{w}q^2 - \Omega_r^2} & q > \dfrac{v_0}{\sqrt{-v_e\mathrm{w}}}R^{-1} \end{cases} \quad \text{(S15)}$$

Here, we use the relationship $R = v_0/\Omega_r$. The instability point, at which the system begins to become unstable, is defined as $\kappa_2^{max} = 0$ for the second branch in Eq. (S15). Mathematically, it is identical to constrain the equation $D_e q^2 + \sqrt{-v_e\mathrm{w}q^2 - \Omega_r^2} = 0$ to have only one positive solution q^*, which leads to

$$v_e \mathrm{w} = -2\Omega_r D_e \tag{S16}$$

$$q^* = \sqrt{\frac{\Omega_r}{D_e}} \tag{S17}$$

These two equations can be further simplified by introducing the critical packing fraction $\phi_c \simeq 0.32$ for the system with $\Omega_\mathrm{r} = 0$ ($R = \infty$). From Eq. (S16) we will have w=0 at $\phi = \phi_c$, which leads to $\zeta = -v_0 \phi_c^{-1} \sigma^2 \pi/8$. The expressions for the critical packing fraction ϕ^* and critical q^* as a function of R then can be written as

$$\left(\frac{\phi^*}{\phi_c} - 1\right)\left(2 - \frac{\phi^*}{\phi_c}\right) = \frac{8D_e}{v_0 R} \tag{S18}$$

$$q^* = \sqrt{\frac{v_0}{RD_e}} \tag{S19}$$

Section S3. Calculation of $S^o(q)$ for system with CMC

Based on Eq. (12) in the main text, the density fluctuation of the effective particles in the Fourier space can be written as

$$\delta\rho^{\mathrm{o}}_{\mathbf{q},\omega} = \frac{-q^2\sqrt{\overline{\rho}}\eta_{\mathbf{q},\omega}}{i\omega + D_e q^2} \qquad \left(q \ll \frac{2\pi}{R}\right) \tag{S20}$$

Here we assumed $D_e^{\mathrm{o}} = D_e$. By defining

$$\int dt = \int_{-\infty}^{+\infty} dt = \lim_{\tau_{max}\to\infty} \int_{-\frac{\tau_{max}}{2}}^{+\frac{\tau_{max}}{2}} dt \tag{S21}$$

we can write down the dynamic structure factor as

$$\begin{aligned}
S^{\mathrm{o}}(k,\omega) &= \frac{1}{2\pi\tau_{max}N}\left\langle \delta\rho^{\mathrm{o}}_{\mathbf{q},\omega}\delta\rho^{\mathrm{o}*}_{\mathbf{q},\omega}\right\rangle \\
&= \left(\frac{q^4\overline{\rho}}{\omega^2 + D_e^2 q^4}\right)\frac{1}{2\pi\tau_{max}N}\left\langle \eta_{\mathbf{q},\omega}\eta^*_{\mathbf{q},\omega}\right\rangle \\
&= \left(\frac{q^4\overline{\rho}}{\omega^2 + D_e^2 q^4}\right)\frac{1}{2\pi\tau_{max}N}\left\langle \int d\mathbf{r} e^{-i\mathbf{q}\cdot\mathbf{r}} \int dt e^{-i\omega t}\eta(\mathbf{r},t) \int d\mathbf{r}' e^{i\mathbf{q}\cdot\mathbf{r}'} \int dt' e^{i\omega t'}\eta(\mathbf{r}',t')\right\rangle \\
&= \left(\frac{q^4\overline{\rho}}{\omega^2 + D_e^2 q^4}\right)\frac{1}{2\pi\tau_{max}N}\int d\mathbf{r}\int d\mathbf{r}' e^{-i\mathbf{q}\cdot(\mathbf{r}-\mathbf{r}')}\int dt \int dt' e^{-i\omega(t-t')}\langle \eta(\mathbf{r},t)\eta(\mathbf{r}',t')\rangle \\
&= \left(\frac{q^4\overline{\rho}}{\omega^2 + D_e^2 q^4}\right)\frac{1}{2\pi\tau_{max}N}\int d\mathbf{r}\int d\mathbf{r}' e^{-i\mathbf{q}\cdot(\mathbf{r}-\mathbf{r}')}\int dt \int dt' e^{-i\omega(t-t')} A^2 \delta(\mathbf{r}-\mathbf{r}')\delta(t-t') \\
&= \left(\frac{q^4\overline{\rho}}{\omega^2 + D_e^2 q^4}\right)\frac{1}{2\pi\tau_{max}N}\int d\mathbf{r}\int dt A^2 \\
&= \left(\frac{q^4}{\omega^2 + D_e^2 q^4}\right)\frac{A^2}{2\pi} \qquad \left(q \ll \frac{2\pi}{R}\right)
\end{aligned} \tag{S22}$$

The static structure factor can be obtained by doing the integration

$$\begin{aligned} S^{o}(\mathbf{q}) &= \int_{-\infty}^{\infty} S^{o}(k,\omega)d\omega \\ &= \frac{A^2q^4}{2\pi}\int_{-\infty}^{\infty}\frac{1}{\omega^2+D_e^2q^4}d\omega \\ &= \frac{A^2q^4}{2\pi}\frac{\pi}{D_eq^2} \\ &= \frac{A^2}{2D_e}q^2 \qquad \left(q \ll \frac{2\pi}{R}\right) \end{aligned} \quad (S23)$$

Section S4. Effect of thermal noise on $S^{o}(q)$

For isolated circle active particles, at finite temperature T Brownian motion contributes a R dependent effective self-diffusion constant (*25*)

$$\begin{aligned} D_{therm} &= D_t + \frac{\gamma_t^{-2}F_p^2D_r}{2(D_r^2+\Omega_r^2)} \\ &\simeq D_t + \frac{v_0^2D_r}{2\Omega_r^2} \\ &= k_BT\gamma_t^{-1}\left(1+\frac{R^2}{\sigma^2}\right) \end{aligned} \quad (S24)$$

Here, we assume the orientational relaxation time D_r^{-1} due to the rotational Brownian motion is much longer than the circling period of active particles. At finite temperature T, the noise term $\xi(t)$ in the diffusion equation Eq. (11) of the main content should be a combination of the chaotic noise $\eta(t)$ and the thermal noise, which conserves the number of particles but not the center of mass

$$\xi(t) = \sqrt{\bar{\rho}}\nabla\cdot[\nabla\eta(t)+\mathbf{f}(t)] \quad (S25)$$

where $\mathbf{f} = [f_x, f_y]$ represents the thermal noise which obeys $\langle f_i(\mathbf{r},t)f_j(\mathbf{r}',t')\rangle = 2D_{therm}\delta_{ij}\delta(\mathbf{r}-\mathbf{r}')\delta(t-t')$. Here we assume that the thermal noise is a first-order perturbation on the chaotic noise $\eta(t)$, which leads to the decoupling of these two noise sources: $\langle f_i(\mathbf{r},t)\eta(\mathbf{r}',t')\rangle = 0$. Density fluctuations at large length scale in the Fourier space is

$$\delta\rho^{o}_{\mathbf{q},\omega} = -\frac{\sqrt{\bar{\rho}}\left(q^2\eta_{\mathbf{q},\omega}+i\mathbf{q}\cdot\mathbf{f}_{q,\omega}\right)}{i\omega+D_eq^2} \qquad \left(q \ll \frac{2\pi}{R}\right) \quad (S26)$$

The dynamic structure factor becomes

$$\begin{aligned} S^{o}(k,\omega) &= \frac{1}{2\pi\tau_{max}N}\left\langle\delta\rho^{o}_{\mathbf{q},\omega}\delta\rho^{o*}_{\mathbf{q},\omega}\right\rangle \\ &= \left(\frac{\bar{\rho}}{\omega^2+D_e^2q^4}\right)\frac{1}{2\pi\tau_{max}N}\left[q^4\left\langle\eta_{\mathbf{q},\omega}\eta^*_{\mathbf{q},\omega}\right\rangle + q_x^2\left\langle f_{x,\mathbf{q},\omega}f^*_{x,\mathbf{q},\omega}\right\rangle + q_y^2\left\langle f_{y,\mathbf{q},\omega}f^*_{y,\mathbf{q},\omega}\right\rangle\right] \\ &= \left(\frac{\bar{\rho}}{\omega^2+D_e^2q^4}\right)\frac{1}{2\pi\tau_{max}N}\left[q^4\left\langle\eta_{\mathbf{q},\omega}\eta^*_{\mathbf{q},\omega}\right\rangle + q^2\left\langle f_{i,\mathbf{q},\omega}f^*_{i,\mathbf{q},\omega}\right\rangle\right] \\ &= \frac{A^2q^4+2D_{therm}q^2}{2\pi(\omega^2+D_e^2q^4)} \qquad \left(q \ll \frac{2\pi}{R}\right) \end{aligned} \quad (S27)$$

And finally the static structure factor is

$$
\begin{aligned}
S^{\mathrm{o}}(\mathbf{q}) &= \int_{-\infty}^{\infty} S^{\mathrm{o}}(k,\omega)d\omega \\
&= \frac{D_{therm}}{D_e} + \frac{A^2}{2D_e} \\
&= \frac{k_B T}{\gamma_t D_e}\left(1+\frac{R^2}{\sigma^2}\right) + \frac{A^2}{2D_e} q^2 \\
&= \frac{v_0 \sigma}{D_e}\left(1+\frac{R^2}{\sigma^2}\right) T_R + \frac{A^2}{2D_e} q^2 \qquad \left(q \ll \frac{2\pi}{R}\right) \qquad \text{(S28)}
\end{aligned}
$$

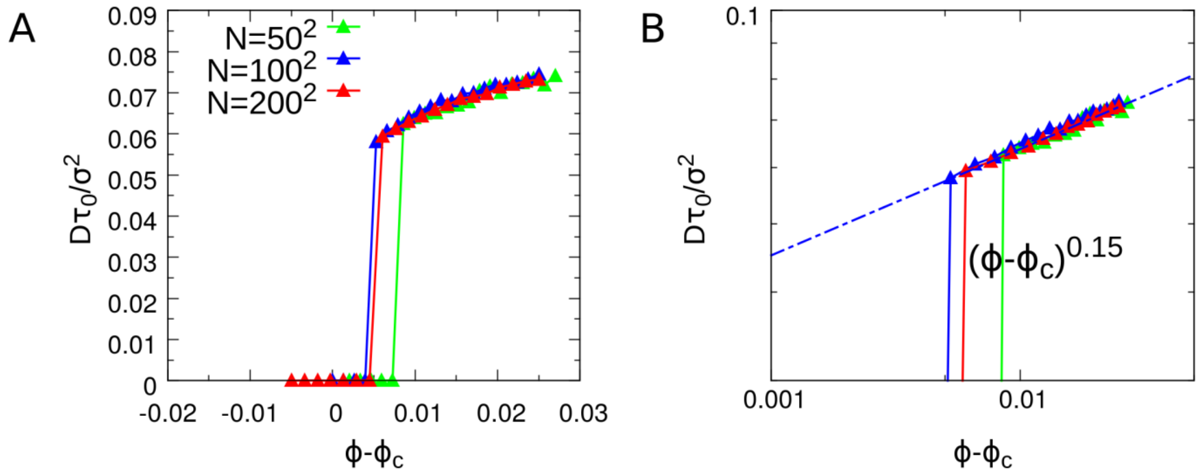

Fig. S1. Finite-size effect on the long-time diffusion coefficient *D* as a function of packing fraction ϕ for systems with *R* = 1.75σ. The critical packing fraction obtained from the fitting is $\phi_c = 0.194, 0.195, 0.195$ for $N = 50^2, 100^2, 200^2$ respectively.

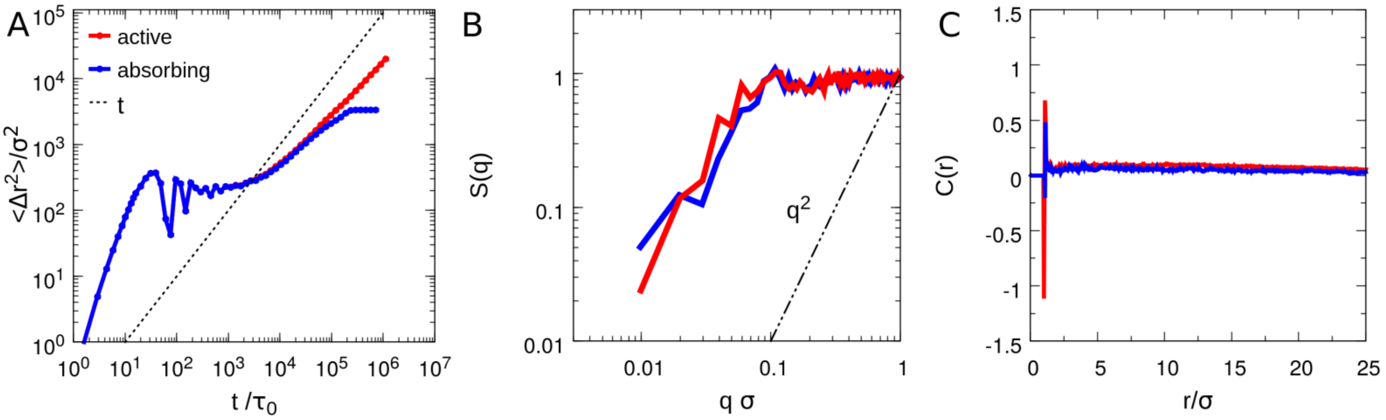

Fig. S2. Structural comparison of active and absorbing state at *R* = 10σ near the critical point ϕ_c = 0.0194. (**A**): MSD; (**B**): Structure factor $S(q)$ where the q^2 hyperuniform scaling is found for the active state; (C): Orientational correlation function $C(\mathrm{r})$. The absorbing state is at $\phi = 0.0191$ and the active state is at $\phi = 0.0195$.

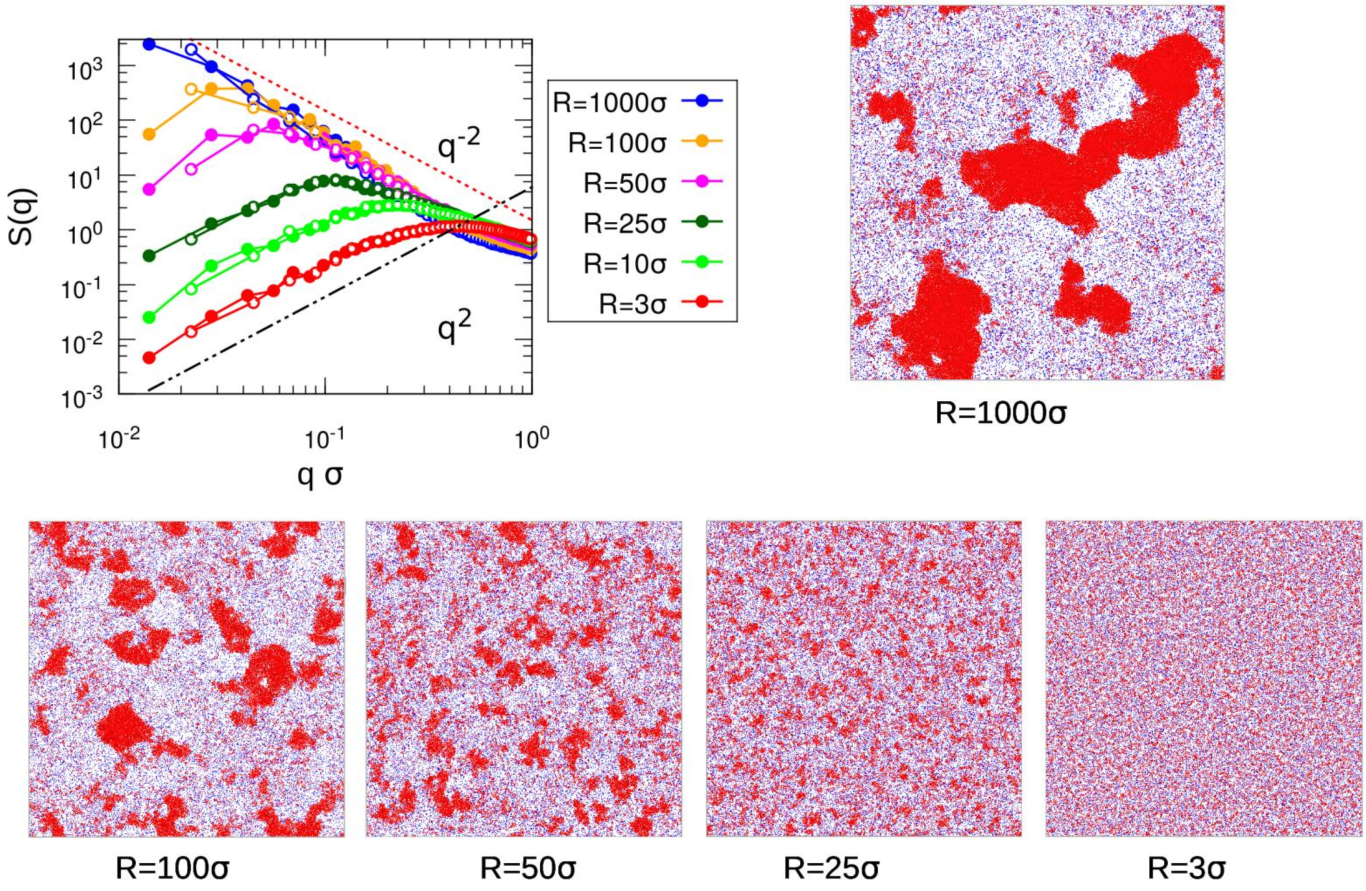

Fig. S3. Structure factor $S(q)$ for large systems with different R at $\phi = 0.4$ and $T_R = 0$. Solid symbols are for $N = 102{,}400$ system, while open symbols are for $N = 40{,}000$. The snapshots show typical configurations for the $N = 102{,}400$ system with different R. Particles who have a distance less than 1σ with their nearest neighbor are denoted as red otherwise blue.

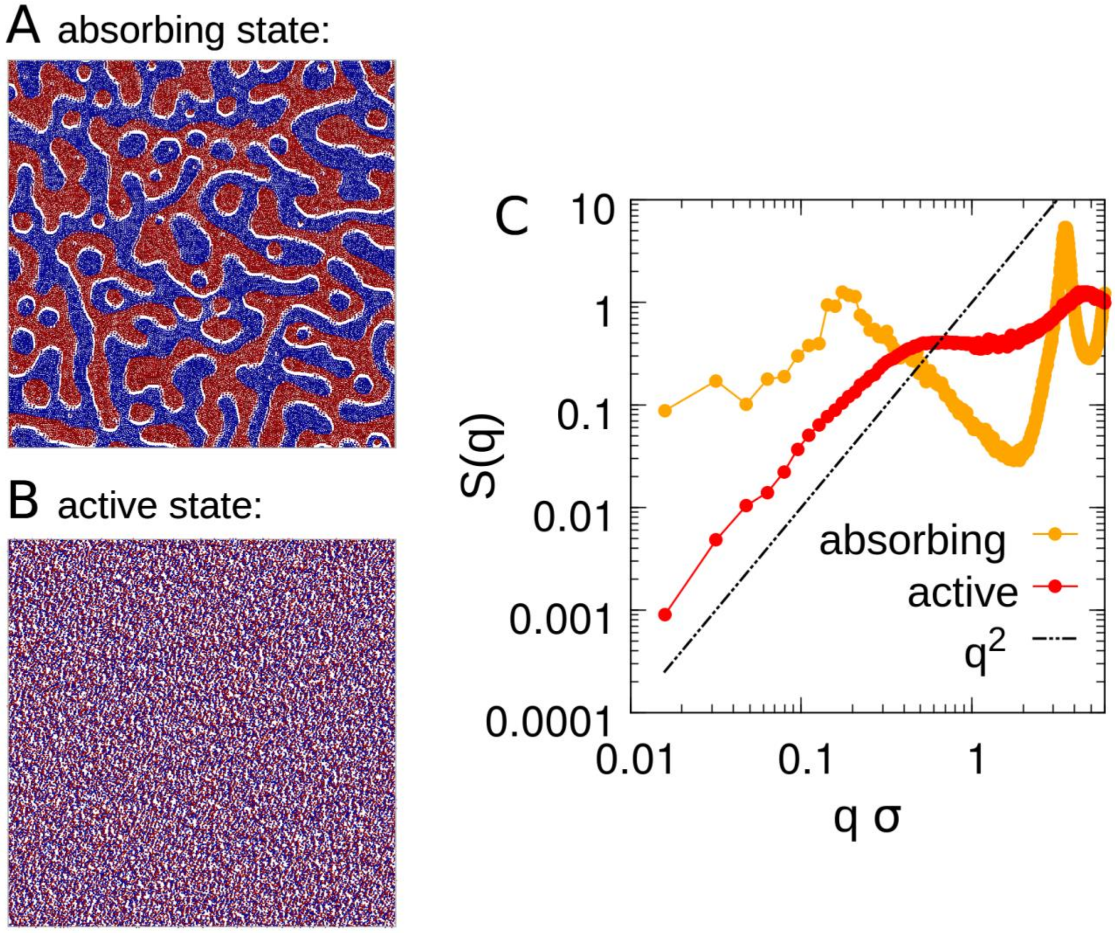

Fig. S4. Hyperunifomity in an experimentally realizable system (*26*) with bimodal circling-phase distribution. (**A**, **B**): Configurations of the phase-separated absorbing state with $R = 1\sigma$ and the active mixing (lane) state with $R = 3\sigma$. (**C**): Structure factor for the two states where the q^2 hyperuniform scaling is found for the active state. The packing fraction for both cases is $\phi = 0.2$.
